# Periodontitis‐compromised dental pulp stem cells secrete extracellular vesicles carrying miRNA‐378a promote local angiogenesis by targeting Sufu to activate the Hedgehog/Gli1 signalling

**DOI:** 10.1111/cpr.13026

**Published:** 2021-03-23

**Authors:** Huan Zhou, Xuan Li, Rui‐Xin Wu, Xiao‐Tao He, Ying An, Xin‐Yue Xu, Hai‐Hua Sun, Li‐An Wu, Fa‐Ming Chen

**Affiliations:** ^1^ Department of Periodontology School of Stomatology State Key Laboratory of Military Stomatology National Clinical Research Center for Oral Diseases and Shaanxi Engineering Research Center for Dental Materials and Advanced Manufacture Fourth Military Medical University Xi’an China; ^2^ Shaanxi Key Laboratory of Free Radical Biology and Medicine The Ministry of Education Key Laboratory of Hazard Assessment and Control in Special Operational Environments Fourth Military Medical University Xi’an China

**Keywords:** angiogenesis, dental pulp stem cells, extracellular vesicles, periodontitis, Sufu

## Abstract

**Objectives:**

Previously, our investigations demonstrated robust pro‐angiogenic potentials of extracellular vesicles secreted by periodontitis‐compromised dental pulp stem cells (P‐EVs) when compared to those from healthy DPSCs (H‐EVs), but the underlying mechanism remains unknown.

**Materials and methods:**

Here, circulating microRNAs (miRNAs) specifically found in P‐EVs (compared with H‐EVs) were identified by Agilent miRNA microarray analysis, and the roles of the candidate miRNA in P‐EV‐enhanced cell angiogenesis were confirmed by cell transfection and RNA interference methods. Next, the direct binding affinity between the candidate miRNA and its target gene was evaluated by luciferase reporter assay. CCK‐8, transwell/scratch wound healing and tube formation assays were established to investigate the proliferation, migration, and tube formation abilities of endothelial cells (ECs). Western blot was employed to measure the protein levels of Hedgehog/Gli1 signalling pathway components and angiogenesis‐related factors.

**Results:**

The angiogenesis‐related miRNA miR‐378a was found to be enriched in P‐EVs, and its role in P‐EV‐enhanced cell angiogenesis was confirmed, wherein Sufu was identified as a downstream target gene of miR‐378a. Functionally, silencing of Sufu stimulated EC proliferation, migration and tube formation by activating Hedgehog/Gli1 signalling. Further, we found that incubation with P‐EVs enabled the transmission of P‐EV‐contained miR‐378a to ECs. Subsequently, the expressions of Sufu, Gli1 and vascular endothelial growth factor in ECs were significantly influenced by P‐EV‐mediated miR‐378a transmission.

**Conclusions:**

These data suggest that P‐EVs carrying miR‐378a promote EC angiogenesis by downregulating Sufu to activate the Hedgehog/Gli1 signalling pathway. Our findings reveal a crucial role for EV‐derived miR‐378a in cell angiogenesis and hence offer a new target for modifying stem cells and their secreted EVs to enhance vessel regenerative potential.

## Graphical abstract

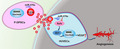

## 1 INTRODUCTION

Dental pulp stem cells (DPSCs) from periodontitis‐compromised teeth (P‐DPSCs), as a more easily assessable cell population isolated from traditionally discarded tissues, exhibited (to a certain degree) regenerative potential similar to that of DPSCs arising from healthy pulp tissues (H‐DPSCs) based on our previous study.^1^ More importantly, P‐DPSCs can be envisioned as a new and attractive source of mesenchymal stem cells (MSCs) for tissue engineering, as millions of teeth are extracted annually due to periodontitis, and these discarded teeth may be an inexhaustible cell source for research and therapeutic purposes, rather than as mere medical and biological waste.

Given that extracellular vesicles (EVs) secreted by H‐DPSCs (H‐EVs) have been proven to facilitate vascularization and vessel formation,^2^ we further explored the pro‐angiogenic potential of EVs secreted by P‐DPSCs (P‐EVs). To this end, we found that P‐EVs profoundly enhanced the proliferation, migration and angiogenesis of endothelial cells (ECs) in vitro and were able to accelerate cutaneous wound healing and promote vessel formation in vivo. Importantly, P‐EVs demonstrated an enhanced pro‐angiogenic capacity compared to H‐EVs secreted by donor‐matched H‐DPSCs, but the underlying mechanism remains unexplored.^3^

EVs, the cell‐secreted nanovesicles, are lipid bilayer structures carrying a spectrum of bioactive molecules including but not limited to proteins, mRNAs, lipids and microRNAs (miRNAs); these molecules, which contribute to intercellular communication, can be transferred to their co‐existing cells or tissues to exert biological functions.^4^, ^5^ Among them, miRNAs have been characterized as the most vital cargoes in EVs; miRNAs are small endogenous non‐coding RNAs (21‐23 nucleotides in length) that function as regulators of mRNA expression in most cell types, and can directly interact with complementary regions in the 3′ untranslated region (UTR) of target mRNAs, thereby reducing the stability or inhibiting the translation of specific mRNAs.^6^, ^7^ Increasing evidence indicates that specific miRNAs can be selectively packaged into EVs and then delivered to proximal or distal cells to modulate a variety of physiological and pathological processes, including angiogenesis. In this context, various miRNAs such as miR‐29a,^8^ miR‐423‐5p^9^ and miR‐181b‐5p^10^ contained in EVs of various cell types have shown pro‐angiogenic activity and hence are able to promote angiogenesis.

Therefore, in this study, we hypothesized that the pro‐angiogenic potential of P‐EVs resulted from the specific angiogenesis‐related miRNAs they contained and that those miRNAs could be transferred to co‐existing ECs to promote cell proliferation, migration and angiogenesis. To test this hypothesis, we screened the differentially expressed miRNAs between P‐EVs and H‐EVs and then identified the downstream target genes of the identified miRNAs to further interrogate the potential signalling pathways underlying miRNA‐mediated EC angiogenesis. A deeper understanding of the mechanism behind P‐EV‐enhanced cell angiogenesis offers a new target for modifying stem cells and their secreted EVs towards vessel regeneration in tissue engineering and regenerative medicine.

## 2 MATERIALS AND METHODS

### 2.1 Isolation and identification of EVs

The isolation of EVs was performed with ultracentrifugation methods as reported previously.^11^ Initially, the collected sample medium was centrifuged at 300 × g (10 minutes), 2000 × g (10 minutes) and 10,000 × g (30 minutes) in order to eliminate cells and debris. Then, the sample supernatant was centrifuged at 100,000 × g (70 minutes), after which the pellets were washed with phosphate‐buffered saline (PBS; Corning, NY, USA) under the same conditions (100,000 × g for 70 minutes) to obtain pure EVs. To ascertain the EV identification, Western blot was performed to detect EV‐specific biomarkers (see Section 2.9), transmission electron microscopy (TEM; Hitachi, Tokyo, Japan) was utilized to observe EV morphology, and nanoparticle tracking analysis (NTA; NanoSight 300; Malvern Instruments, Malvern, UK) was employed to assess P‐EV size and concentration.

### 2.2 Identification of differentially expressed miRNAs in P‐EVs and H‐EVs (bioinformatics analysis)

The miRNA molecular changes in P‐EVs (compared to H‐EVs) were identified by analysis of an Agilent miRNA microarray. Initially, TRIzol Reagent (Invitrogen) was used to extract total RNA from EVs (P‐EVs and H‐EVs). Then, the RNA quality and quantity were measured by a NanoDrop ND‐1000, and RNA integrity was assessed by standard denaturing agarose gel electrophoresis. RNA labelling and array hybridization were carried out according to the Agilent miRNA Microarray System with the miRNA Complete Labeling and Hyb Kit protocol (Agilent Technology). Briefly, total miRNA from P‐EVs and H‐EVs was labelled with Cy3‐pCp under the action of the T4 RNA ligase. After that, the labelled cRNA was subjected to inspissation, desiccation, and then dissolved in water. Then, 1 μg of each labelled cRNA was fragmented by adding 11 μL 10 × Blocking Agent and 2.2 μL of 25 × Fragmentation Buffer, and then heated for 30 minutes at 60°C. Then, 55 μL 2 × GE Hybridization buffer was added to dilute the labelled cRNA, and the hybridization solution (100 μL) was dispensed into the gasket slide, which was assembled within the gene expression microarray slide. The slides were incubated in an Agilent Hybridization Oven for 17 hours at 65°C. In the end, the hybridized arrays were washed, fixed and scanned using an Agilent Microarray Scanner (part number, G2505C). Agilent Feature Extraction software (version 11.0.1.1) and the GeneSpring GX v12.1 software package (Agilent Technologies) were utilized to analyse and process the data. Subsequently, miRNA‐specific quantitative real‐time PCR(qRT‐PCR) analysis (see Section 2.8) was employed to validate the results of differentially expressed miRNAs in P‐EVs and H‐EVs.

### 2.3 The role of miR‐378a in the angiogenesis of ECs (cell transfection)

miR‐378a gene transfection was performed to verify whether miR‐378a was involved in the angiogenesis of ECs. Briefly, human umbilical vein ECs (CRL‐1730; American Type Culture Collection, Rockefeller) were cultured in 12‐well culture plates and transfected with either mimic‐negative control (mimic‐NC) or miR‐378a mimic (100 μM, GenePharma) using Lipofectamine 3000 Transfection Reagent (Thermo Fisher Scientific) in strict accordance with the manufacturer's specifications; ECs without transfection were used as the blank control (Control group). Cell proliferation, migration and tube formation on Matrigel assays were performed at 48 hours after cell transfection (see Section 2.7).

### 2.4 Identification of the target gene of miR‐378a

The potential genes (as well as the binding sites) targeted by miR‐378a were predicted with the biological prediction website TargetScan. Among the predicted candidates, suppressor of fused (Sufu), which has been proven to be closely related to angiogenesis,^9^ was chosen as the potential target gene of miR‐378a for further study. To further test and verify the relationship between Sufu and miR‐378a, a series of assays in terms of luciferase reporter, qRT‐PCR and Western blot were performed.

Initially, a luciferase reporter assay was conducted to verify the direct binding affinity between miR‐378a and Sufu. Briefly, luciferase reporter plasmids (pGL3 vector; GenePharma) with Sufu 3′‐UTRs containing mutant or putative binding sites of miR‐378a were constructed. ECs were then transfected with the Sufu 3′‐UTR wild‐type (WT) or mutant (MUT) plasmids in the presence of miR‐378a mimic or mimic‐NC in strict accordance with the manufacturer's specifications. After 48 hours, ECs were lysed, and the luciferase activities were detected with a dual‐luciferase reporter assay kit (Yeasen, Shanghai, China) according to the protocols.

Meanwhile, qRT‐PCR and Western blot were performed to validate the relationship between Sufu and miR‐378a. In brief, after reaching 80% confluence, ECs were transfected with mimic‐NC, miR‐378a mimic (100 μM), inhibitor‐NC and miR‐378a inhibitor (200 μM) using Lipofectamine 3000 in strict accordance with the manufacturer's specifications, and then, the expression of Sufu was measured by qRT‐PCR and Western blot (see Sections 2.8 & 2.9). Oligonucleotide sequences for miR‐378a are shown in Table S2.

### 2.5 Elucidation of the role of Sufu in the angiogenesis of ECs

To verify the function of Sufu in inhibiting EC angiogenesis, we assessed whether silencing of Sufu could enhance the angiogenesis of ECs. For this purpose, three small interfering RNAs (siRNAs) targeting Sufu (si‐Sufu #1, 2 and 3; 100 μM; GenePharma) were used to knockdown the expression of Sufu in ECs (oligonucleotide sequences for si‐Sufu are shown in Table S2.). ECs were transfected with si‐Sufu #1, 2 or 3 according to the instructions of the manufacturers, and the silencing efficiencies (after 48 hours of transfection) were verified by qRT‐PCR; the siRNA that resulted in the lowest Sufu expression level in ECs was selected to investigate the effects of Sufu silencing on EC angiogenesis via cell proliferation, migration and tube formation on Matrigel assays (see Section 2.7), wherein ECs transfected with siRNA negative control (si‐NC) and ECs without transfection were used as the negative control (si‐NC group) and blank control (Control group), respectively. Given that Sufu is a negative regulator of the Hedgehog/Glioma‐associated oncogene homologue 1(Gli1) signalling pathway, we further detected whether silencing Sufu could increase Gli1 expression level in ECs (Western blot assay, see Section 2.9).

### 2.6 The role of Hedgehog/Gli1 signalling in EC angiogenesis induced by P‐EV‐mediated transmission of miR‐378a

#### 2.6.1 Confirmation of P‐EV‐mediated transmission of miR‐378a to ECs

To verify whether P‐EVs can deliver miR‐378a to ECs, we used a Cy3‐labelled miR‐378a mimic (GenePharma) to modify P‐EVs according to the previously reported methods^12^, ^13^ and then investigated how miR‐378a mimic‐modified P‐EVs can be taken up by co‐cultured ECs. In brief, the obtained EV pellet was re‐suspended in sterile PBS, and then mixed with Exo‐Fect solution (Exo‐Fect Kit, EXFT20A‐1; System Biosciences) and Cy3‐labelled miR‐378a mimic (Cy3 dye alone without conjugation to miR‐378a mimic was used as the control) in a tube. Next, the tube was incubated at 37°C for 10 minutes in a shaker and then immediately placed on ice; the adding of ExoQuick‐TC reagent was used to stop the reaction. Following centrifugation at 14 000 rpm for 3 minutes, the EV pellet modified by miR‐378a mimic (or the Cy3 dye control) was then re‐suspended in PBS and diluent C. Subsequently, the solution was mixed with PKH67 reagent (PKH67 kit; Sigma‐Aldrich) and incubated at room temperature for 4 minutes. Finally, the PKH67‐labelled P‐EVs were washed with PBS at 100,000 × g (70 minutes) before being added to EC cultures. Following incubation for 6 hours at 37°C, 4',6‐diamidino‐2‐phenylindole (DAPI; Heart) was used to label EC nuclei, and the cellular uptake of miR‐378a mimic‐modified P‐EVs by ECs was observed with a confocal imaging system (Carl Zeiss).

#### 2.6.2 Effects of P‐EV‐mediated miR‐378a transmission on EC angiogenesis

ECs were respectively treated with 100 μg/mL P‐EVs (without modification) or 100 μg/mL P‐EVs that were separately modified with mimic‐NC, miR‐378a mimic, inhibitor‐NC and miR‐378a inhibitor using the Exo‐Fect Kit, and then divided into the following groups: P‐EVs (Control group); P‐EVs^mimic‐NC^; P‐EVs^miR‐378a mimic^; P‐EVs^inhibitor‐NC^ and P‐EVs^miR‐378a inhibitor^. After the treatment, cell proliferation, migration and tube formation on Matrigel assays (See Section 2.7) were carried out to interrogate the EC angiogenesis abilities. In addition, Western blot (See Section 2.9) was conducted to detect the protein expression of Hedgehog/Gli1 signalling components (Sufu, Gli1) and vascular endothelial growth factor (VEGF) in ECs.

#### 2.6.3 Effect of Hedgehog/Gli1 signalling inhibition on EC angiogenesis induced by P‐EV‐mediated transmission of miR‐378a

To identify whether the Hedgehog/Gli1 signalling pathway is involved in EC angiogenesis arising from P‐EV‐mediated miR‐378a transmission, we further investigated how Hedgehog/Gli1 signalling inhibition can affect the angiogenesis abilities of P‐EVs^miR‐378a mimic^‐treated ECs. Here, the specific Hedgehog/Gli1 signalling inhibitor GANT61 (20 μM; #HY‐13901; MedChemExpress, New Jersey, USA) was used, and the cellular responses (proliferation, migration and tube formation; See Section 2.7) along with changes in VEGF expression (See Section 2.9) were recorded.

### 2.7 Cell angiogenesis assays

#### 2.7.1 Cell proliferation assay

The Cell Counting Kit‐8 (CCK‐8) assay was employed to determine the EC viability changes in response to various treatments.^14^ Briefly, ECs were inoculated into 96‐well plates at a concentration of 5 × 10^3^ cells per well. After incubation for 24, 48, 72, 96 and 120 hours, CCK‐8 reagent (10 μL; Dojindo) was added to each well, and ECs were incubated for another 3 hours. Then, the optical density (OD) value at 450 nm was recorded with an Infinite M200 PRO microplate reader (TECAN, Männedorf, Switzerland).

#### 2.7.2 Cell migration assay

A transwell migration assay was employed to investigate changes in the EC migration ability in response to various treatments as previously reported.^15^ In brief, 2 × 10^4^ (cells/well) ECs were plated into the apical chambers of transwell culture plates (24‐well; 8 μm pore‐sized filters; Corning) in 200 μL serum‐free medium, while the basolateral chamber was filled with 600 μL culture medium containing 20% FBS. Following an 8‐h incubation, the non‐migrated ECs were wiped off gently with a cotton swab, while the migrated cells on the bottom membrane surface were fixed with paraformaldehyde (4%) and stained with crystal violet solution (Heart) at room temperature for 30 minutes. Subsequently, the number of migrating cells was counted in five randomly selected microscopic fields. A scratch wound healing assay was also carried out to measure the EC migration ability as previously reported.^16^ More specifically, pre‐treated ECs were plated into 12‐well plates at a concentration of 2 × 10^5^ cells/well (Corning) and allowed to adhere overnight; then, a sterile 200 μL pipette tip was utilized to make a vertical scratch. To remove the dislodged cells, the wells were gently washed twice with PBS. Subsequently, the images of migrated cells were captured under a microscope at 0, 6 and 12 hours. ImageJ software was used to quantify the outcomes.

#### 2.7.3 Tube formation assay on Matrigel

For the tube formation assay, cold Matrigel (50 μL/well; Corning, #356234) was inoculated into the 96‐well plates (Corning) and incubated for 30 minutes at 37°C^17^; then, 2 × 10^4^ ECs were plated on the Matrigel pre‐coated well. After 6 hour of incubation, the images of tube‐like structures were photographed, and tube formation potential in terms of total branching points and total tube length was quantified with ImageJ software in five randomly selected fields.

### 2.8 qRT‐PCR analysis

For the gene expression analysis, total RNA was extracted with the MiniBEST Universal RNA Extraction Kit (TaKaRa, Tokyo, Japan) or the miRNA Mini Kit (QIAGEN, Dusseldorf, Germany) in strict accordance with the instructions of the manufacturers. PrimeScript™ RT reagent (TaKaRa) was used for mRNA reverse transcription, and a miRNA First Strand cDNA Synthesis Tailing Reaction Kit (B532451, Sangon Biotech, Shanghai, China) was utilized for miRNA reverse transcription. After that, the TB Green™ Premix Ex Taq™ II kit (TaKaRa) was utilized to perform qRT‐PCR on a Real‐Time PCR Detection System (Bio‐Rad). U6 was utilized to normalize the miRNA expression levels. For the analysis of miRNA expression in EVs, samples were spiked with cel‐miR‐39 (miRB0000010‐3‐1, RIOBO) to control inter‐sample variability as described in previous studies.^18^ For mRNA analysis, β‐actin was employed to normalize the levels of genes of interest. The sequences of all primers used in the present study are shown in Table S1.

### 2.9 Western blot analysis

Equal amounts of protein from cells (or EVs) were separated via sodium dodecyl sulphate‐polyacrylamide gel electrophoresis (SDS‐PAGE) and then transferred to PVDF membranes (Millipore). After blocking in 5% non‐fat milk with Tris‐buffered saline Tween‐20 (TBST, Heart), the membrane strips were incubated with primary antibodies in terms of anti‐β‐actin (1:1000; Proteintech, Rosemont, USA; #60008‐1‐lg), anti‐ALIX (1:1000; Cell Signaling Technology, Danvers, MA, USA; #2171), anti‐CD9 (1:1000; Cell Signaling Technology; #13174), anti‐CD81 (1:1000; Abcam, Cambridge, Britain; ab109201), anti‐VEGF (1:1000; Abcam; ab46154), anti‐Sufu (1:1000; Cell Signaling Technology; #2520) and anti‐Gli1 (1:1000; Cell Signaling Technology; #3538) overnight at 4°C. Then, the membranes were washed using TBST three times and incubated with HRP‐conjugated secondary antibodies (1:5000, Proteintech; SA00001‐1 or SA00001‐2) for 2 h at room temperature. Subsequently, the protein bands were detected with the chemiluminescent detection reagent (Zeta Life) and analysed with ImageJ software. The expression levels of proteins were quantified based on the β‐actin level.

### 2.10 Statistical analysis

All the experiments were performed at least in triplicate, and the data are presented as the mean ± standard deviation (SD). Paired t test analysis was employed for two‐group comparisons; one‐way analysis of variance (ANOVA) and Tukey's post‐test were utilized for multiple‐group comparisons. *P* <.05 was judged to be indicative of statistical significance.

## 3 RESULTS

### 3.1 miR‐378a differentially expressed in P‐EVs and H‐EVs

**FIGURE 1:**
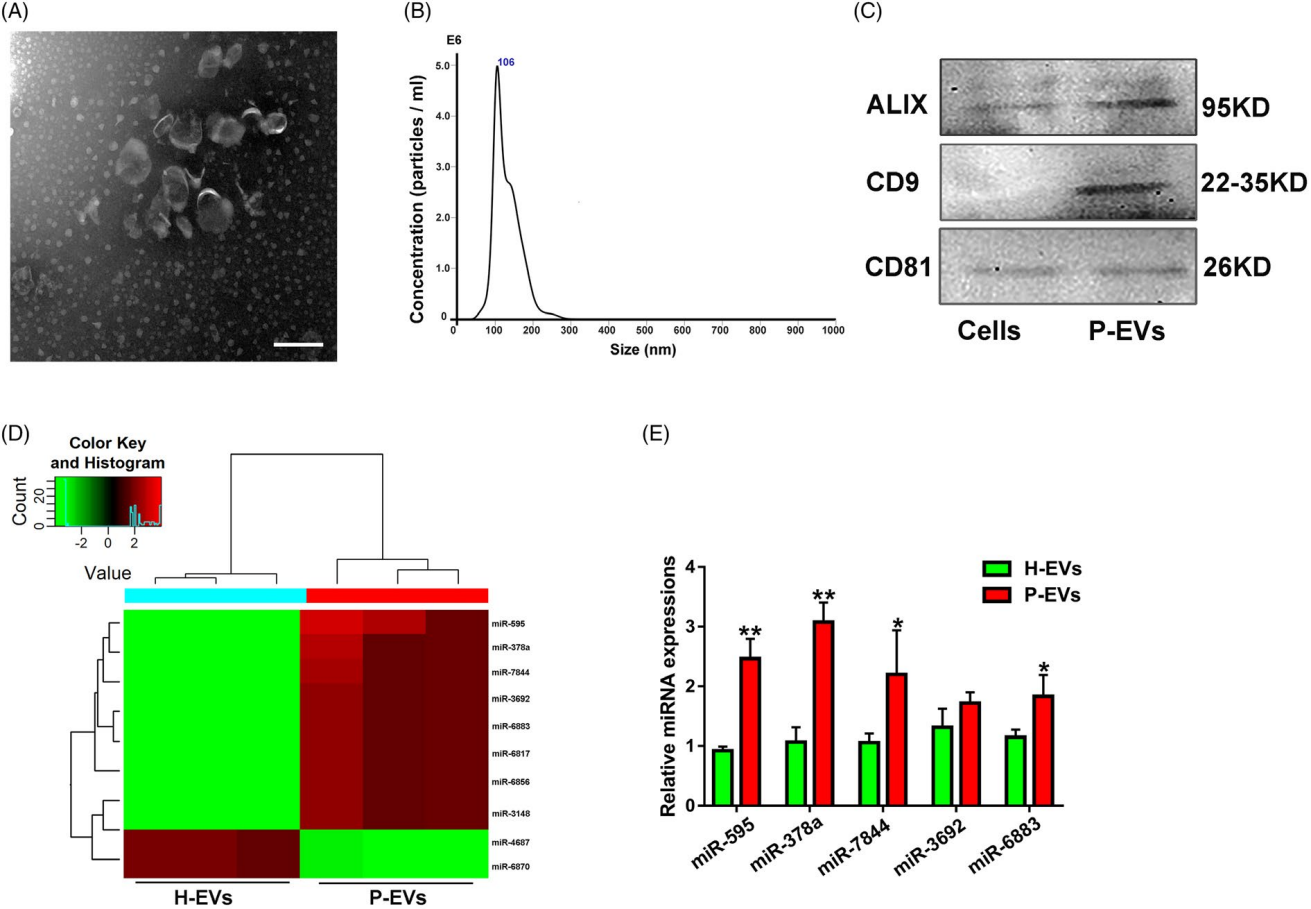
Identification of an angiogenesis‐related miRNA (miR‐378a) differentially expressed in P‐EVs and H‐EVs. (A) Morphology of P‐EVs under transmission electron microscopy. (B) The size and concentration of P‐EVs were identified by nanoparticle analysis. (C) EV surface markers of ALIX, CD9 and CD81 detected by Western blot. (D) Heat map of differentially expressed miRNAs in P‐EVs and H‐EVs (fold change > 1.2, *P* <.05), wherein red colour represents a relative high expression level of the related miRNAs while green colour represents a relative low expression level of the related miRNAs. (E) Validation of the differentially expressed miRNAs screened from the Heat map in P‐EVs and H‐EVs by qRT‐PCR (n = 3); *P <.05 and **P <.01 vs. H‐EVs

P‐EVs were isolated from P‐DPSCs (for the isolation and identification of P‐DPSCs, see Figure S1 and Figure S2) by ultracentrifugation methods, and then, their identification was confirmed by electron microscopy, Western blot and NTA. As shown in Figure 1A, the obtained particles were bilayer membrane vesicles, coinciding with the general characteristics of EVs. Additionally, the diameters of P‐EVs mostly ranged from 50 to 200 nm based on the NTA results (Figure 1B). Furthermore, as expected, P‐EVs expressed the specific markers in terms of ALIX, CD9 and CD81, as evidenced by Western blot (Figure 1C), further confirming their EV identity.

miRNAs are small non‐coding RNAs that act as post‐transcriptional regulators by inhibiting the protein translation. More importantly, miRNAs can be selectively packed into EVs, and when EVs are ingested by target cells, their contained miRNAs may induce transcriptomic changes, further modulating various physiological and pathological processes, including angiogenesis.^19^ In this context, we hypothesized that P‐EVs exert their pro‐angiogenic functions by transferring specific miRNAs; thus, we analysed the global expression of miRNAs in P‐EVs and further compared and explored the differentially expressed miRNAs in P‐EVs compared with H‐EVs through Agilent miRNA microarray analysis. According to the data, P‐EVs had a specific miRNA signature that was quite different from that of H‐EVs (Figure 1D). Among the uncovered abundant miRNAs, miR‐378a, which was highly expressed and more enriched in P‐EVs than in H‐EVs, emerged as a candidate of interest because it has been reported to be involved in angiogenesis.^20^ In addition, we validated this result by miRNA‐specific qRT‐PCR analysis (Figure 1E). According to these findings, we speculated that miR‐378a may play a crucial role in the pro‐angiogenic effect of P‐EVs, and subsequently focused on miR‐378a in further studies.

### 3.2 Overexpression of miR‐378a in ECs promotes cell angiogenesis

**FIGURE 2:**
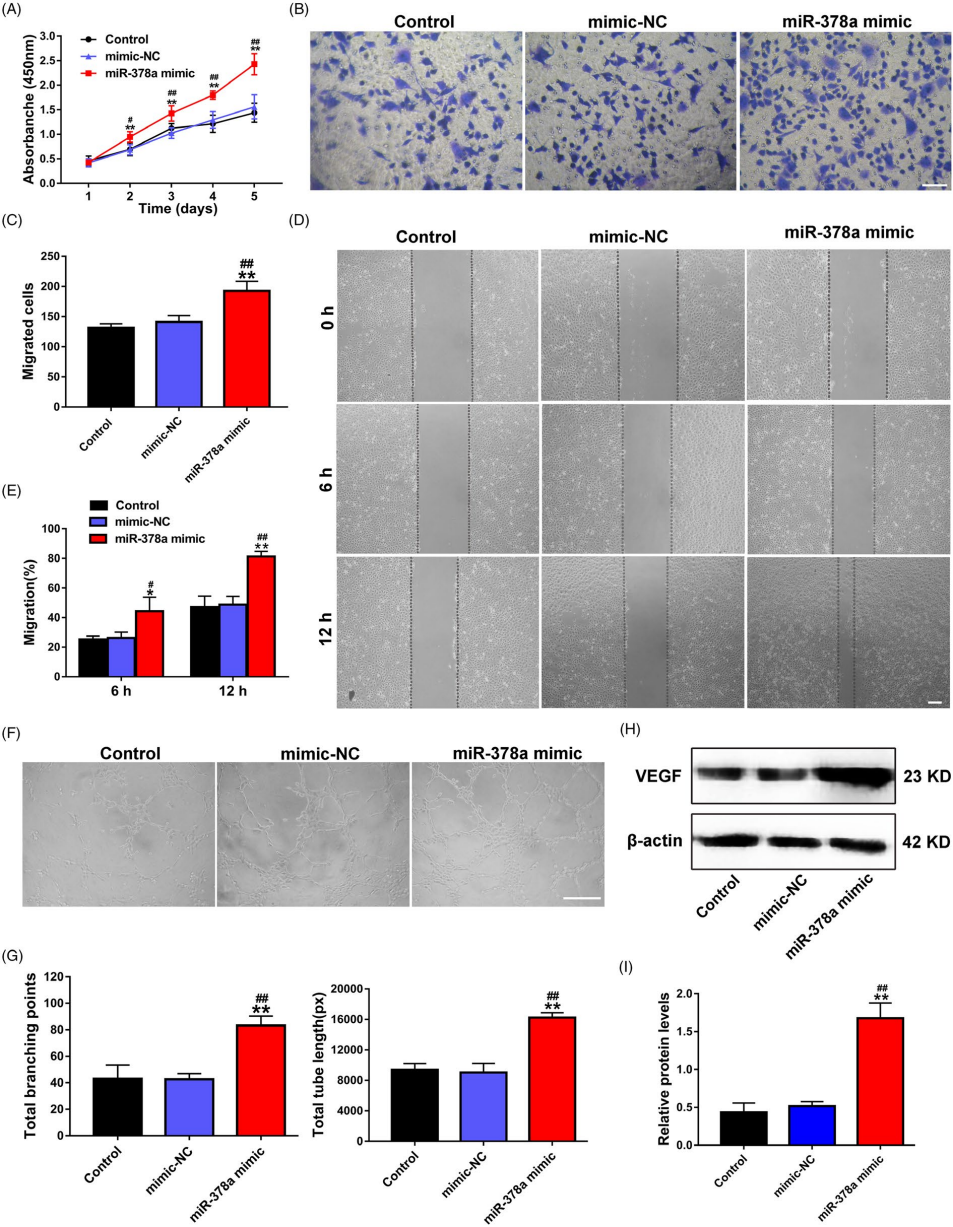
Overexpression of miR‐378a in ECs promotes cell proliferation, migration and angiogenesis. (A) The proliferation of ECs treated with PBS, mimic‐NC and miR‐378a mimic was tested by CCK‐8 assay (n = 3). (B) The migration of ECs treated with PBS, mimic‐NC and miR‐378a mimic was detected by transwell assay (scale bar, 100 μm). (C) Quantitative analysis of the migrated cells in B (n = 5). (D) The migration of ECs treated with PBS, mimic‐NC and miR‐378a mimic was measured by scratch wound assay (scale bar, 200 μm). (E) Quantitative analysis of the migration rates in D (n = 5). (F) Representative images of the tube formation assay in ECs treated with PBS, mimic‐NC and miR‐378a mimic (scale bar, 200 μm). (G) Quantitative analyses of the total branching points and total tube length in F (n = 3). (H) Detection of the protein level of VEGF in ECs by Western blot. (I) Quantitative analysis of the relative protein expression in H (n = 3). *P <.05, **P <.01 vs. the Control group; ^#^ *P* <.05, ^##^ *P* <.01 vs. the mimic‐NC group

The synthesized miR‐378a mimic was transfected directly into ECs to determine whether miR‐378a could indeed promote EC angiogenesis. After overexpression of miR‐378a in ECs, a series of angiogenesis‐related assays, including cell proliferation, migration and tube formation assays, were performed. In terms of proliferation assay, the CCK‐8 assay showed that the EC proliferation rate in the miR‐378a mimic group was significantly higher in relative to that of the Control and mimic‐NC groups (Figure 2A). Additionally, compared with the Control and mimic‐NC groups, ECs in the miR‐378a mimic group exhibited a stronger migration ability, as demonstrated by the transwell and scratch wound healing assay results (Figure 2B‐E). More importantly, tube formation on Matrigel assay showed that the total tube length and number of branches in ECs transfected with miR‐378a mimic were significantly increased than those in the other two groups (Figure 2F‐G). In addition, the protein expression level of VEGF was substantially upregulated in the miR‐378a mimic group compared with the Control and mimic‐NC groups (Figure 2H‐I).

### 3.3 Validation of Sufu as a downstream target gene of miR‐378a

**FIGURE 3:**
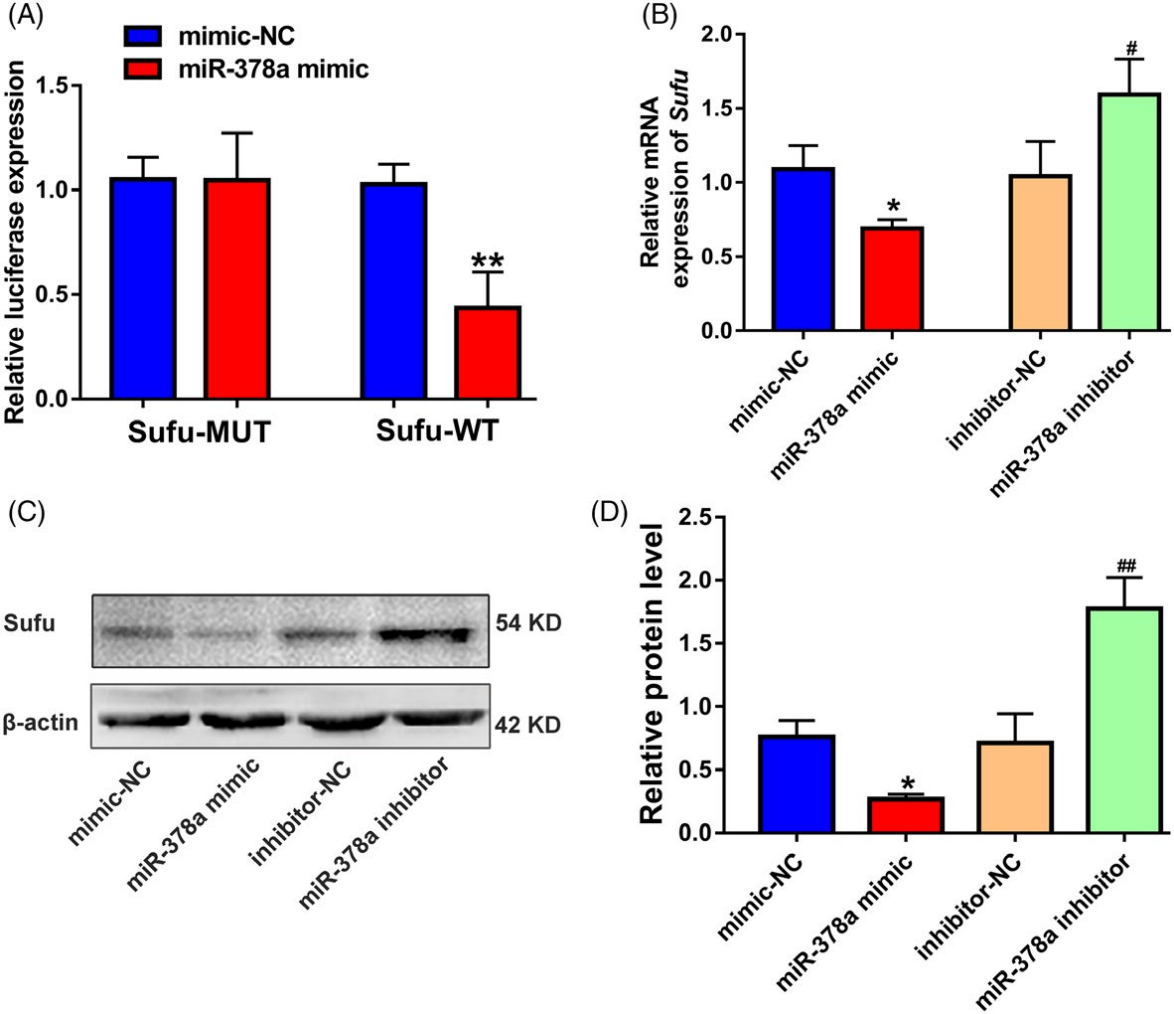
Identification of Sufu as a downstream target gene of miR‐378a. (A) The relative luciferase activity of luciferase reports with wild‐type or mutant Sufu was determined in ECs, which were transfected with mimic‐NC or miR‐378a mimic (n = 3). (B) The mRNA expression of *Sufu* in ECs after transfection with mimic‐NC, miR‐378a mimic, inhibitor‐NC and miR‐378a inhibitor was determined by qRT‐PCR (n = 3). (C) The protein expression of Sufu in ECs detected using Western blot. (D) Quantitative analysis of the relative protein expression in C (n = 3). *P <.05, **P <.01 vs. the mimic‐NC group; ^#^ *P* <.05, ^##^ *P* <.01 vs. the inhibitor‐NC group

The potential downstream target genes of miR‐378a were initially predicted using an online prediction website, with the aim of revealing the molecular mechanism underlying the pro‐angiogenic activity of miR‐378a. Among the predicted candidates, Sufu emerged as a potential target gene (of miR‐378a) of interest, as it has been proven to suppress angiogenesis.^9^ More importantly, according to the computational analysis, the miR‐378a‐targeted site in the 3′‐UTR of Sufu is highly conserved among vertebrates and is partially complementary to miR‐378a. To further verify the accuracy of the prediction, a dual‐luciferase assay was established. ECs were co‐transfected with mimic‐NC and Sufu‐MUT, mimic‐NC and Sufu‐WT, miR‐378a mimic and Sufu‐MUT, or miR‐378a mimic and Sufu‐WT. As shown in Figure 3A, compared with the mimic‐NC group, the Sufu‐WT group exhibited a significant decrease in luciferase activity with miR‐378a mimic treatment, but this treatment had no significant effect on the luciferase activity of the Sufu‐MUT group. In addition, as presented in Figure 3B‐D, both qRT‐PCR and Western blot revealed that the expression (mRNA and protein) of Sufu were downregulated in ECs treated with miR‐378a mimic, whereas Sufu mRNA and protein expression were upregulated in ECs treated with the miR‐378a inhibitor.

### 3.4 Silencing of Sufu promotes angiogenesis and increases Gli1 expression level in ECs

**FIGURE 4:**
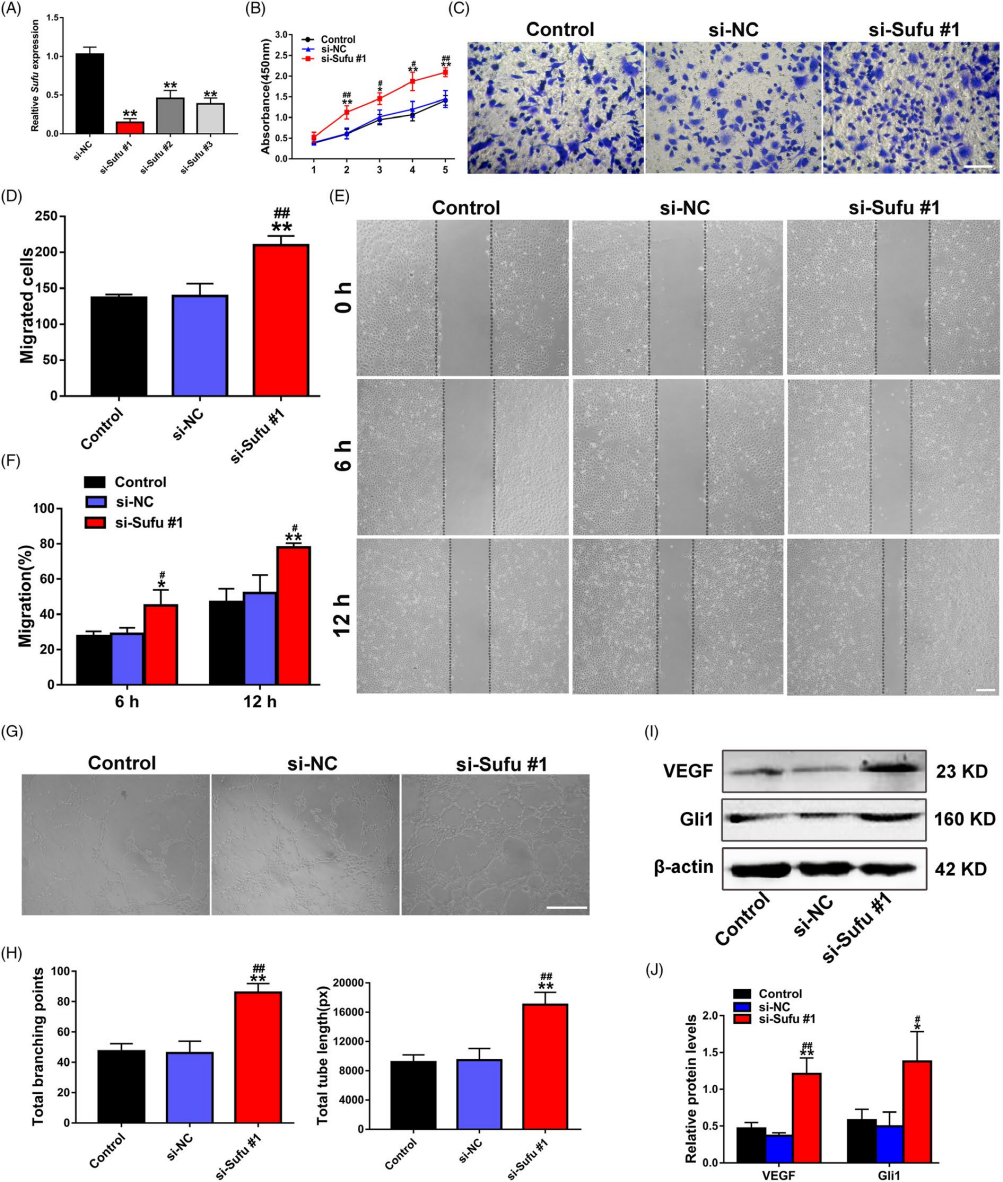
Silencing of Sufu promotes EC proliferation, migration and angiogenesis via Hedgehog/Gli1 signalling activation. (A) The inhibitory efficiency of the siRNAs targeting Sufu was verified by qRT‐PCR. (B) The proliferation of ECs treated with PBS, si‐NC and si‐Sufu #1 was detected by CCK‐8 assay (n = 3). (C) The migration of ECs treated with PBS, si‐NC and si‐Sufu #1 was determined by transwell assay (scale bar, 100 μm). (D) Quantitative analysis of the migrated cells in C (n = 5). (E) The migration of ECs treated with PBS, si‐NC and si‐Sufu #1 was detected by scratch wound assay (scale bar, 200 μm). (F) Quantitative analysis of the migration rates in E (n = 5). (G) Representative images of the tube formation assay in ECs treated with PBS, si‐NC and si‐Sufu #1 (scale bar, 200 μm). (H) Quantitative analyses of the total branching points and total tube length in G (n = 3). (I) Detection of the protein level of VEGF in ECs by Western blot. (J) Quantitative analysis of the relative protein expression in I (n = 3). *P <.05, **P <.01 vs. the Control group; ^#^ *P* <.05, ^##^ *P* <.01 vs. the si‐NC group

We next asked whether silencing of Sufu could enhance the angiogenesis of ECs. Three siRNAs (si‐Sufu #1, 2 and 3) were utilized to downregulate the Sufu expression in ECs, respectively. Then, the silencing efficiency of these siRNAs was verified by qRT‐PCR; the most effective siRNA in terms of si‐Sufu #1 was chosen for the further functional studies (Figure 4A). The results of CCK‐8 (Figure 4B), transwell (Figure 4C, D), scratch wound assay (Figure 4E, F) and tube formation on Matrigel assays (Figure 4G, H) collectively revealed that Sufu silencing can significantly enhance the proliferation, migration and angiogenesis potential of ECs. In addition, si‐Sufu #1 treatment significantly upregulated VEGF protein expression level based on the Western blot results (Figure 4I, J). To detect whether Hedgehog/Gli1 signalling was involved in the EC angiogenesis, Western blot assay was established, and the results showed an increase in the Gli1 protein expression level in ECs after si‐Sufu #1 treatment. Quantitative analysis revealed that Sufu silencing significantly upregulated the Gli1 protein expression level (Figure 4I, J).

### 3.5 Incubation with P‐EVs enables miR‐378a transmission to ECs

**FIGURE 5:**
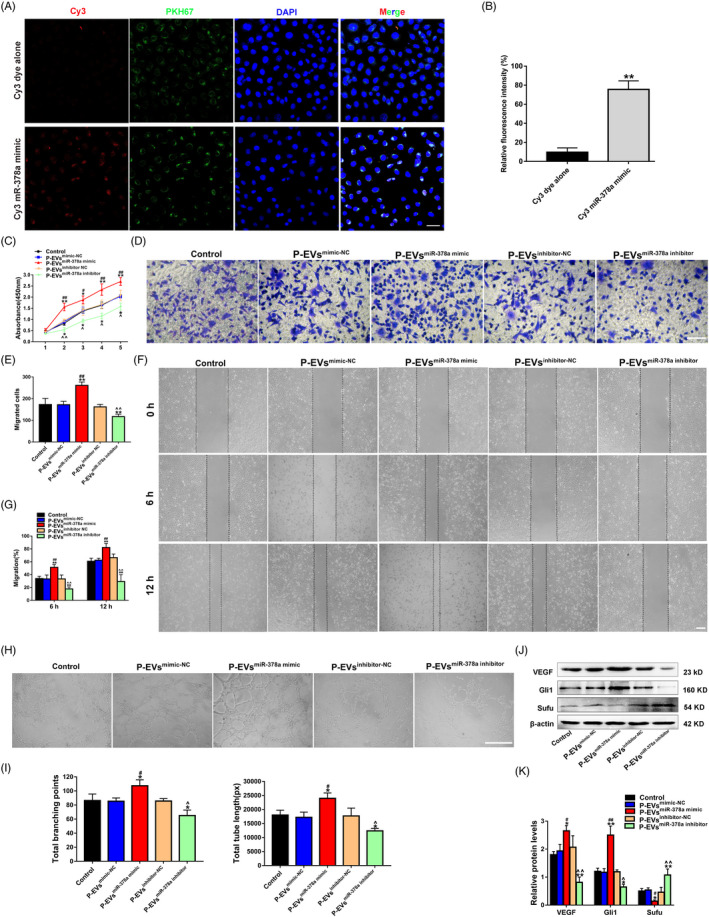
P‐EV‐mediated transmission of miR‐378a promotes EC angiogenesis through activating Hedgehog/Gli1 signalling. (A) The presence of Cy3 fluorescence and PKH67 lipid dye in ECs after adding PKH67 (green) labelled P‐EVs that modified with Cy3‐labelled miR‐378a mimic (red) (the bottom panels). ECs incubated with Cy3 dye alone (not conjugated to miR‐378a mimic) were used as the control (the top panels). Nuclei were stained with DAPI (blue) for counterstaining (scale bar, 50 μm). (B) Quantitative analysis of the fluorescence intensity in A (n = 3, **P <.01 vs. Cy3 dye along). (C) The proliferation of ECs exposed to P‐EVs^mimic‐NC^, P‐EVs^miR‐378a mimic^, P‐EVs^inhibitor‐NC^, P‐EVs^miR‐378a inhibitor^ and an equal volume of PBS was tested by CCK‐8 assay (n = 3). (D) The migration of ECs stimulated by P‐EVs^mimic‐NC^, P‐EVs^miR‐378a mimic^, P‐EVs^inhibitor‐NC^, P‐EVs^miR‐378a inhibitor^ and PBS was detected by transwell assay (scale bar, 100 μm). (E) Quantitative analysis of the migrated cells in D (n = 5). (F) Representative images of the scratch wound assay in ECs treated with P‐EVs^mimic‐NC^, P‐EVs^miR‐378a mimic^, P‐EVs^inhibitor‐NC^, P‐EVs^miR‐378a inhibitor^ and PBS (scale bar, 200 μm). (G) Quantitative analysis of the migration rates in F (n = 3). (H) Representative images of the tube formation assay in ECs treated with P‐EVs^mimic‐NC^, P‐EVs^miR‐378a mimic^, P‐EVs^inhibitor‐NC^, P‐EVs^miR‐378a inhibitor^ and PBS (scale bar, 200 μm). (I) Quantitative analyses of the total branching points and total tube length in H (n = 3). (J) Protein levels of VEGF, Gli1 and Sufu in ECs detected by Western blot. (K) Quantitative analysis of the relative protein expression in J (n = 3). *P <.05, **P <.01 vs. the Control group; ^#^ *P* <.05, ^##^ *P* <.01 vs. the P‐EVs^mimic‐NC^ group; ^^^ *P* <.05, ^^^^ *P* <.01 vs. the P‐EVs^inhibitor‐NC^ group

Subsequently, immunofluorescence assays were established to observe whether P‐EVs can transport miR‐378a to ECs. We transfected P‐EVs with Cy3‐labelled miR‐378a mimic (red) using the commercially available Exo‐Fect Kit and then labelled the P‐EVs with PKH67 (green). As expected, after incubation for 6 hours, both red (staining for the miR‐378a mimic) and green signals (staining for P‐EVs) were observed in the cytoplasm of ECs treated with P‐EVs that modified with Cy3‐labelled miR‐378a mimic (Figure 5A, bottom panels); in control experiments, negligible red signals were observed in ECs (Figure 5A, top panels), and the quantitative results confirmed that the miR‐378a mimic was effectively delivered to ECs by P‐EVs (Figure 5B).

### 3.6 P‐EV‐mediated transmission of miR‐378a promotes EC angiogenesis by activating Hedgehog/Gli1 signalling

A train of assays in terms of proliferation, migration and tube formation were carried out after confirming that P‐EVs can efficiently deliver miR‐378a mimic/inhibitor into ECs (Figure S3). The proliferation ability of ECs was quantified by CCK‐8 assay, which revealed that P‐EVs^miR‐378a mimic^ stimulation resulted in a significant increase in EC proliferation, while the effect was inhibited by P‐EVs^miR‐378a inhibitor^ treatment (Figure 5C). Meanwhile, the results obtained from the wound healing and transwell assay demonstrated that P‐EVs^miR‐378a mimic^ treatment remarkably upregulated the motility of ECs, while the P‐EVs^miR‐378a inhibitor^ exerted the opposite effect (Figure 5D‐G). Moreover, after the P‐EVs^miR‐378a mimic^ treatment, ECs demonstrated a stronger tube formation ability than that in the P‐EVs^mimic‐NC^ group (Figure 5H, I), as shown by quantitative analyses of the total branching points and total tube length. Notably, P‐EVs^miR‐378 inhibitor^ treatment partially reversed the enhanced tube formation trend induced by P‐EV stimulation, as indicated by the quantification of capillary structure formation of ECs (Figure 5H, I). Subsequently, the Hedgehog/Gli1 signalling pathway was evaluated. According to Western blot analysis, P‐EVs^miR‐378a mimic^ induced increases in the protein levels of Gli1 and VEGF and a decrease in the protein level of Sufu, and these effects were opposite to those induced by P‐EVs^miR‐378a inhibitor^ (Figure 5J, K).

**FIGURE 6:**
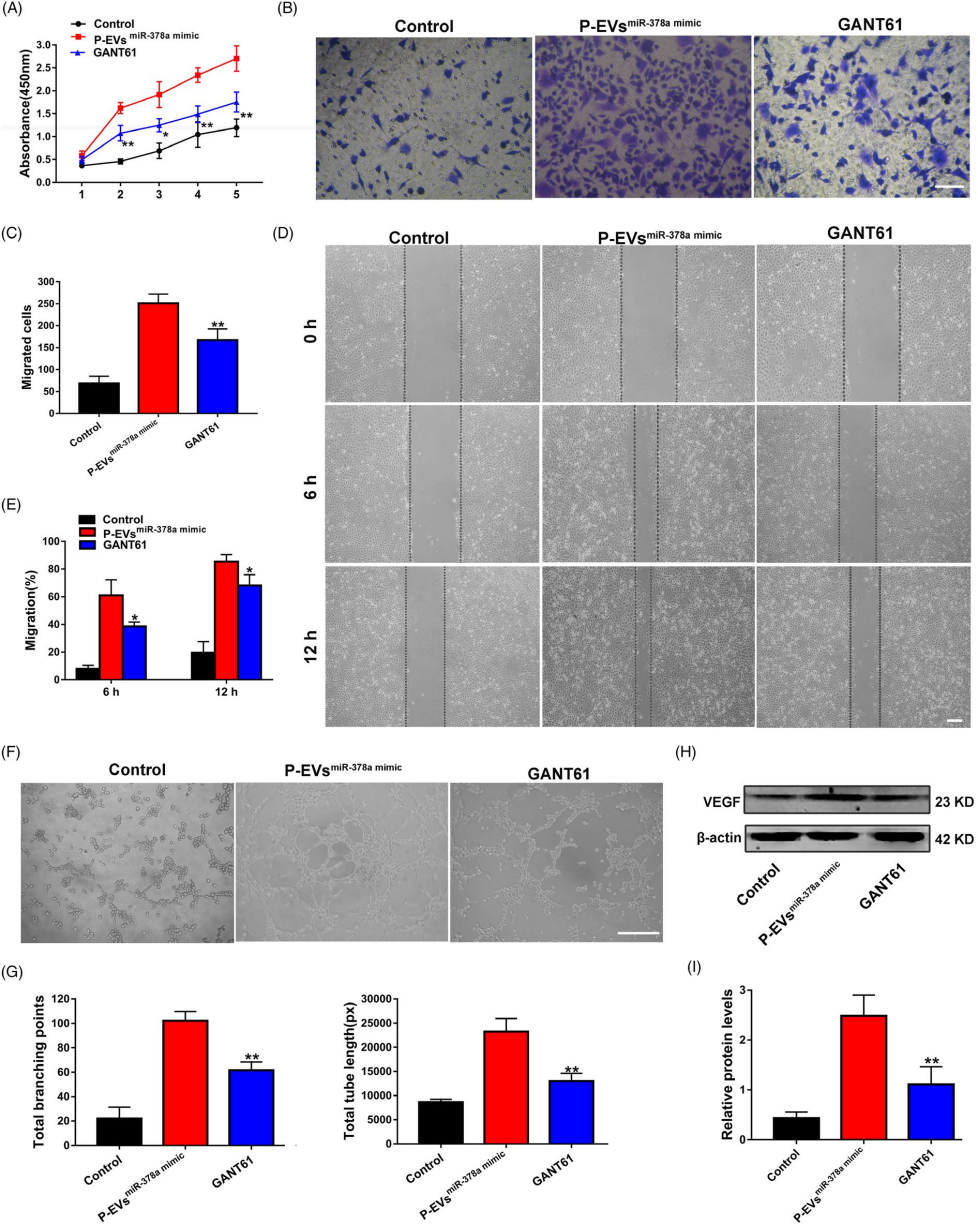
Blockage of Hedgehog/Gli1 signalling reverses the enhanced proliferation, migration and angiogenesis of ECs induced by P‐EVs^miR‐378a mimic^. (A) The proliferation of ECs treated with PBS, P‐EVs^miR‐378a mimic^, and P‐EVs^miR‐378a mimic^ with GANT61 was tested by CCK‐8 assay. (B)The migration of ECs treated with PBS, P‐EVs^miR‐378a mimic^ and P‐EVs^miR‐378a mimic^ with GANT61 was detected by transwell assay (scale bar, 100 μm). (C) Quantitative analysis of the migrated cells in B (n = 5). (D) Representative images of the scratch wound assay in ECs treated with PBS, P‐EVs^miR‐378a mimic^, and P‐EVs^miR‐378a mimic^ with GANT61 (scale bar, 200 μm). (E) Quantitative analysis of the migration rates in D. (F) Representative images of the tube formation assay in ECs (scale bar, 200 μm). (G) Quantitative analyses of the total branching points and total tube length in F (n = 3). (H) Protein levels of VEGF in ECs detected by Western blot. (I) Quantitative analysis of the relative protein expression in H (n = 3). *P <.05, **P <.01 vs. the P‐EVs^miR‐378a mimic^ group

Furthermore, we used the Hedgehog/Gli1 signalling inhibitor GANT61 (an inhibitor of Gli1) to specifically inhibit the Hedgehog signalling pathway in ECs treated with the P‐EVs^miR‐378a mimic^. After treatment with GANT61, the proliferation and migration abilities of ECs were decreased, as determined by CCK‐8 (Figure 6A), Transwell (Figure 6B, C) and wound healing assays (Figure 6D, E). In addition, GANT61 was demonstrated to impair the pro‐angiogenic effect of the P‐EVs^miR‐378a mimic^ in ECs, as represented by decreases in tube length and the number of branch points (Figure 6F, G). Furthermore, the VEGF protein expression level in ECs was downregulated in the GANT61 group compared to the P‐EVs^miR‐378a mimic^ group (Figure 6H, I).

## 4 DISCUSSION

The successful establishment of a highly orchestrated vascular network is essential to tissue engineering, which provides sufficient oxygen and cellular nutrients to sustain adequate growth and regeneration of tissue, especially in injured tissues and organs.^21^ Angiogenesis, defined as the sprouting of new capillaries from pre‐existing blood vessels, is a complex dynamic process regulated by a sequence of molecular and cellular interactions involving cell proliferation, migration, tube formation and maturation into functional blood vessels.^22^ Currently, novel EV‐based therapies have been demonstrated to be very promising strategies for generating functional and successful blood vessels.^14^

Based on the current understanding, stem cells can be recruited to injured or inflamed sites, and their therapeutic effects are mainly mediated by EVs, which are principal paracrine mediators between stem cells and target cells that have attracted extensive attention and investigation.^23^, ^24^ Additionally, emerging knowledge supports the idea that MSC‐released EVs are as effective as their parent stem cells in several tissue regeneration and repair scenarios.^25^, ^26^ In view of this, we isolated P‐EVs and explored their pro‐angiogenic potential and found that P‐EVs can significantly promote EC proliferation, migration and angiogenesis.^3^ However, the exact molecular mechanism remains unknown and needs to be further investigated. In the current study, we determined that miR‐378a is largely responsible for the pro‐angiogenic potential of P‐EVs and can be transferred to ECs, thereby promoting angiogenesis by targeting Sufu to activate Hedgehog/Gli1 signalling.

EV‐based cell‐free therapy offers several advantages that might overcome the obstacles and risks associated with stem cell transplantation approaches.^27^, ^28^ EVs are natural membrane structures containing proteins, lipids, mRNAs and miRNAs and act as messengers by transferring these specific molecules to target cells to contribute to intercellular communication.^29^ Among these molecule types, miRNAs have been studied widely. miRNAs are small non‐coding RNAs that bind to specific mRNAs, thereby inhibiting their translation or reducing their stability. Mounting evidence indicates that a specific class of functional miRNAs can be selectively packaged into EVs and then delivered into target cells to further modulate their activities.^30^, ^31^ In view of this, we hypothesized that P‐EVs might enhance EC angiogenesis by transferring specific miRNAs. To identify the specific miRNAs responsible for the P‐EV‐mediated pro‐angiogenic potential, miRNA profile expression analysis was performed in P‐EVs, and we found that miR‐378a was more enriched in P‐EVs (compared with H‐EVs). Several other studies have demonstrated that miR‐378a has a pro‐angiogenesis effect.^32^, ^33^, ^34^ However, the function of miR‐378a contained in P‐EVs remains largely unknown. Our present study demonstrated that miR‐378a plays a major role in the pro‐angiogenic potential of P‐EVs and that miR‐378a overexpression in ECs leads to increased angiogenesis potential. Previously, Lee et al found that miRNA‐378a can promote cell survival, growth and angiogenesis.^20^ In addition, miR‐378a was reported to be involved in the regulation of VEGF expression.^35^, ^36^ However, the underlying molecular mechanism of miR‐378a‐mediated angiogenesis is poorly understood and deserves more investigation.

To further reveal the underlying mechanism, we performed bioinformatics analysis and identified Sufu, a well‐known negative regulator of the Hedgehog/Gli1 signalling pathway,^37^ as a potential target of miR‐378a. Sufu plays pivotal roles in multiple biological processes, and it has been well documented in various cells that downregulation of Sufu can promote cell proliferation and migration.^38^, ^39^ More importantly, it has been demonstrated that Sufu can suppress angiogenesis.^9^ Additionally, a previous study reported that miR‐378a promotes angiogenesis by targeting Sufu.^20^ In the current study, after confirming that Sufu was the downstream target of miR‐378a, we knocked down Sufu expression in ECs with siRNA and found that the proliferation, migration and angiogenic potential of ECs were markedly augmented. All these effects on ECs induced by si‐Sufu were similar to those observed following exposure to the miR‐378a mimic, which further indicated that the positive effects of P‐EVs on EC function are mediated by miR‐378a‐induced inhibition of Sufu. More importantly, as we expected, ECs treated with P‐EVs^miR‐378a mimic^ (P‐EVs modified with the miR‐378a mimic) demonstrated stronger proliferation, migration and tube formation abilities, while ECs treated with P‐EVs^miR‐378a inhibitor^ (P‐EVs modified with miR‐378a inhibitor) showed the opposite effects.

**FIGURE 7:**
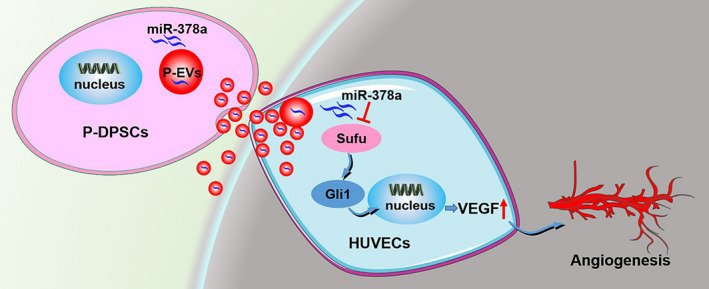
P‐EVs carrying miR‐378a promote the angiogenesis of ECs by downregulating Sufu to activate the Hedgehog/Gli1 signalling pathway

Gli1 is a vital transcription factor of the Hedgehog signalling pathway and has been demonstrated to play a key role in angiogenesis.^40^ Gli1 is also the direct target of Sufu, and previous studies have confirmed that Sufu can interact with Gli1 transcription factors to suppress their nuclear translocation, thereby inhibiting Hedgehog signalling.^41^, ^42^ We then detected Gli1 expression in ECs and found that it was upregulated after Sufu silencing or P‐EVs^miR‐378a mimic^ treatment but downregulated with P‐EVs^miR‐378a inhibitor^ treatment. It has been well demonstrated that the Hedgehog signalling pathway orchestrates various cellular behaviours and biological processes, including cell proliferation, migration, differentiation and survival, and especially the angiogenesis process.^43^, ^44^, ^45^ To further confirm that Hedgehog/Gli1 signalling was involved in EC angiogenesis induced by P‐EV‐mediated transmission of miR‐378a, we treated ECs with GANT61, an inhibitor of Hedgehog/Gli1 signalling; we found that GANT61 treatment partially reversed the enhancement in the proliferation, migration and tube formation induced by P‐EVs^miR‐378a mimic^ treatment and downregulated VEGF expression. Taken together, these results collectively suggest that the pro‐angiogenic effect of P‐EV‐miR‐378a on ECs is exerted, at least in part, via the activation of the Hedgehog/Gli1 signalling pathway (Figure 7). However, considering that there were other miRNAs overexpressed in P‐EVs and only miR‐378a was selected for the present study, we cannot rule out possible contributions from other miRNAs that may act alone or work in a coordinated way in P‐EVs to exert therapeutic effects in the angiogenesis process.

Our study, combined with previously published data show that, as a more readily available cell source in the clinic, P‐DPSCs and other stem cell populations within inflamed tissues, such as human periapical cyst‐mesenchymal stem cells,^46^ can serve as more easily available stem cell resources for regenerative medicine.^47^ Furthermore, the in‐depth study of the role and molecular mechanism of P‐EVs in promoting EC angiogenesis not only expands the research value of stem cells isolated from inflamed tissues, including P‐DPSCs, but also has important significance in promoting their application in vivo. Indeed, EVs, these nano‐sized molecules that physiologically function in cell‐to‐cell cross talk, can be expected to become an important link and breakthrough between basic research and the clinical transformation of inflamed stem cells.^46^ Further characterization is needed to determine the effects of the microenvironment on these inflamed cells and their secreted EVs, because the microenvironment can significantly influence the biological functions of stem cells (DPSCs).^48^ In addition, it remains to be further investigated whether these inflamed cells and their secreted EVs can serve as therapeutic agents for other tissue regeneration scenarios, such as bone regeneration, given that EVs secreted from H‐DPSCs can promote the osteogenesis of stem cells.^49^ From a clinical point of view, these studies and the in‐depth understanding of the underlying molecular mechanism are the premises to employ inflamed cells and their secreted EVs in future tissue engineering applications in vivo, especially diseases with persistent inflammatory stimuli that hinder the success of cell transplantation.

## 5 CONCLUSION

Data obtained from the present study showed that miR‐378a is enriched in P‐EVs and that P‐EVs can transfer their functional miR‐378a to ECs, thereby promoting EC proliferation, migration and angiogenesis by targeting Sufu to activate the Hedgehog/Gli1 signalling pathway. Our data demonstrate a crucial role of miR‐378a derived from the EVs of periodontitis‐compromised dental pulp stem cells in cell angiogenesis and hence offer a new target that could be exploited to modify stem cells and their secreted EVs towards an enhanced angiogenesis/revascularization outcome in regenerative medicine.

## CONFLICT OF INTERESTS

The authors declare that they have no competing interests.

## AUTHORS' CONTRIBUTIONS

HZ, XL, YA and X‐YX contributed to the conception and design of the study. HZ, XL, R‐XW and X‐TH did the job of acquisition, analysis, interpretation of data and manuscript writing. H‐HS, L‐AW and F‐MC contributed to the study conception and design, financial support and manuscript writing. All authors read and approved the final manuscript.

## ETHICS APPROVAL AND CONSENT TO PARTICIPATE

The experimental protocol of this study was approved by the Ethics Committee of the Stomatological Hospital of FMMU (201 203), and informed consent was signed by all the subjects who donated their extracted teeth for cell isolation.

## Supporting information

Fig S1

Fig S2

Fig S3

Supplementary Material

## Data Availability

All data generated or analysed during this study are included in this published article.
