# Supplementary figures and images for: Periodontitis‐compromised dental pulp stem cells secrete extracellular vesicles carrying miRNA‐378a promote local angiogenesis by targeting Sufu to activate the Hedgehog/Gli1 signalling
Source: Cell Prolif. 2021 Mar 23;54(5):e13026. doi: 10.1111/cpr.13026 (PMC8088471; doi:10.1111/cpr.13026)

**Supplemental Results**

**Fig. S1.**


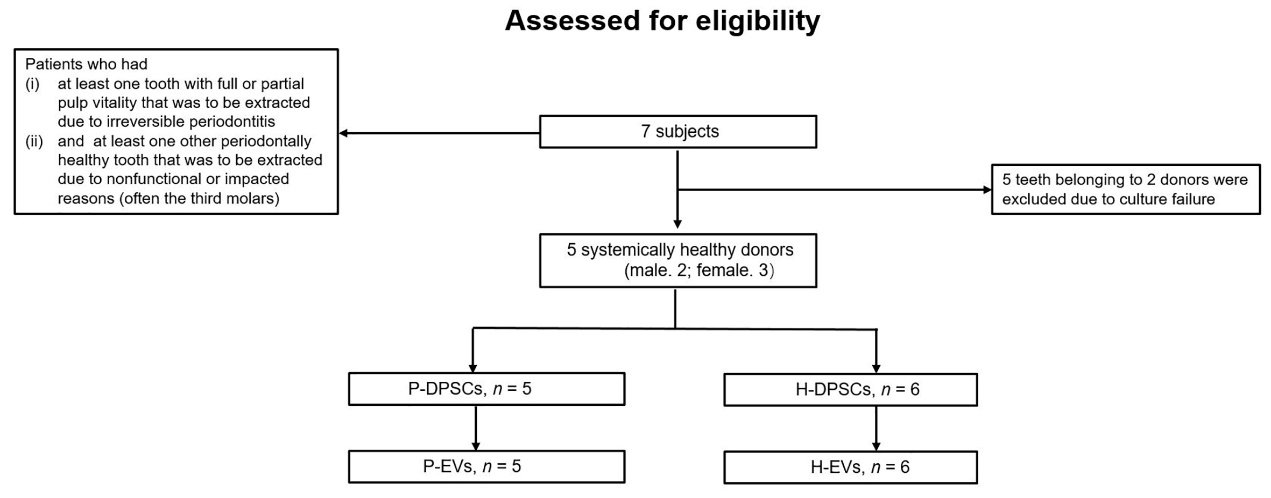


**Fig. S1. Flow chart of the study design.**

Supplement: Supplementary file 1 — Fig S1 [file CPR-54-e13026-s001.docx]
