# Supplementary material for: Periodontitis‐compromised dental pulp stem cells secrete extracellular vesicles carrying miRNA‐378a promote local angiogenesis by targeting Sufu to activate the Hedgehog/Gli1 signalling
Source: Cell Prolif. 2021 Mar 23;54(5):e13026. doi: 10.1111/cpr.13026 (PMC8088471; doi:10.1111/cpr.13026)
Supplement: Supplementary file 2 — Fig S2 [file CPR-54-e13026-s004.docx]

**Supplemental Results**

**Fig. S2.**

**
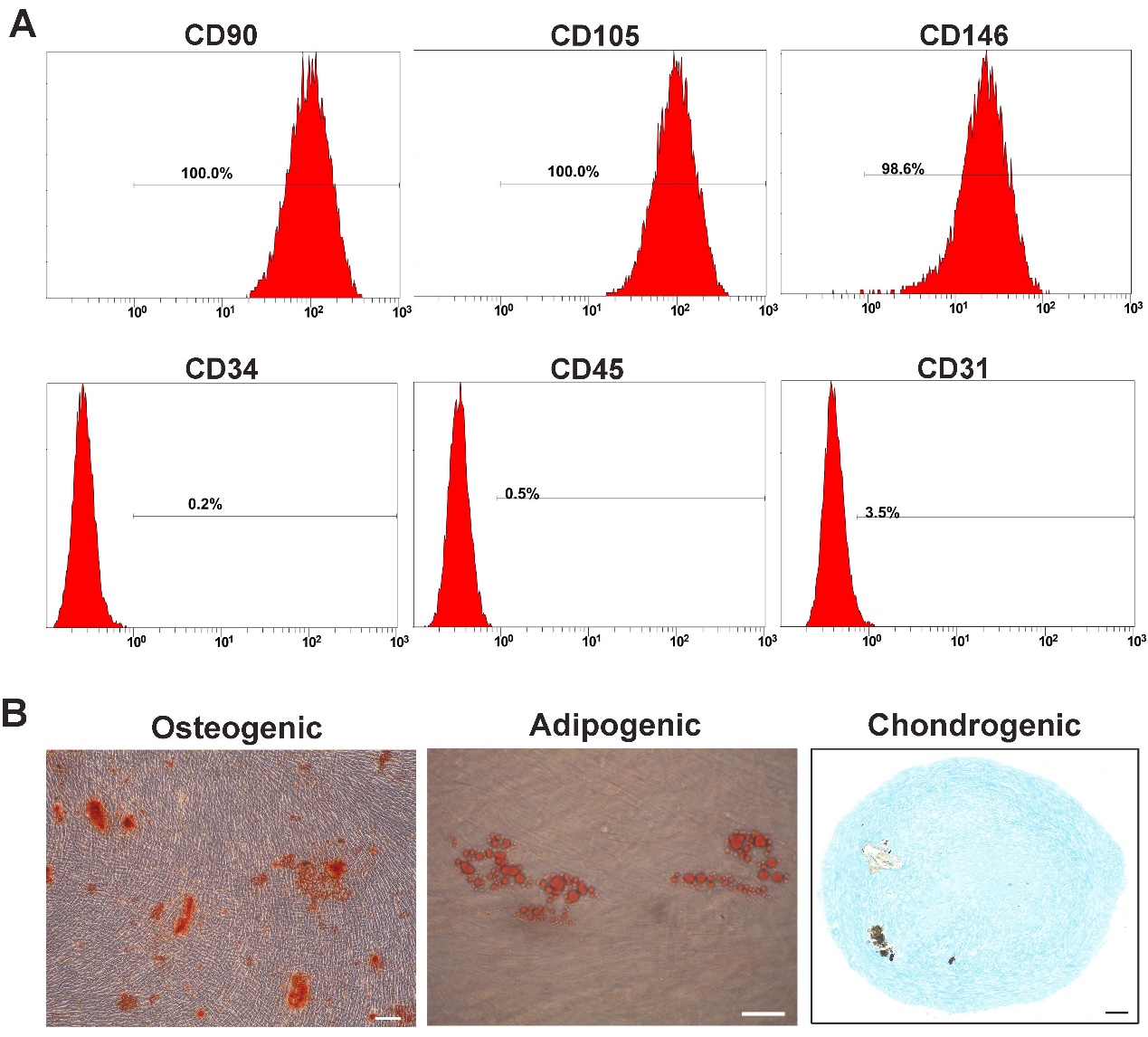
**

**Fig. S2. Isolation and identification of P-DPSCs.** (A) Surface markers of P-DPSCs assayed by flow cytometry: positive for CD90, CD105 and CD146, while negative for CD34, CD45 and CD31. (B) Multiple differentiation potentials of BMMSCs: Alizarin Red S staining for osteogenic differentiation (left; scale bar: 200 μm); Oil Red O staining for adipogenic differentiation (middle; scale bar: 200 μm); Alcian blue staining for chondrogenic differentiation (right; scale bar: 200 μm).
