# Supplementary material for: Periodontitis‐compromised dental pulp stem cells secrete extracellular vesicles carrying miRNA‐378a promote local angiogenesis by targeting Sufu to activate the Hedgehog/Gli1 signalling
Source: Cell Prolif. 2021 Mar 23;54(5):e13026. doi: 10.1111/cpr.13026 (PMC8088471; doi:10.1111/cpr.13026)
Supplement: Supplementary file 3 — Fig S3 [file CPR-54-e13026-s003.docx]

**Supplemental Results**

**Fig. S3.**


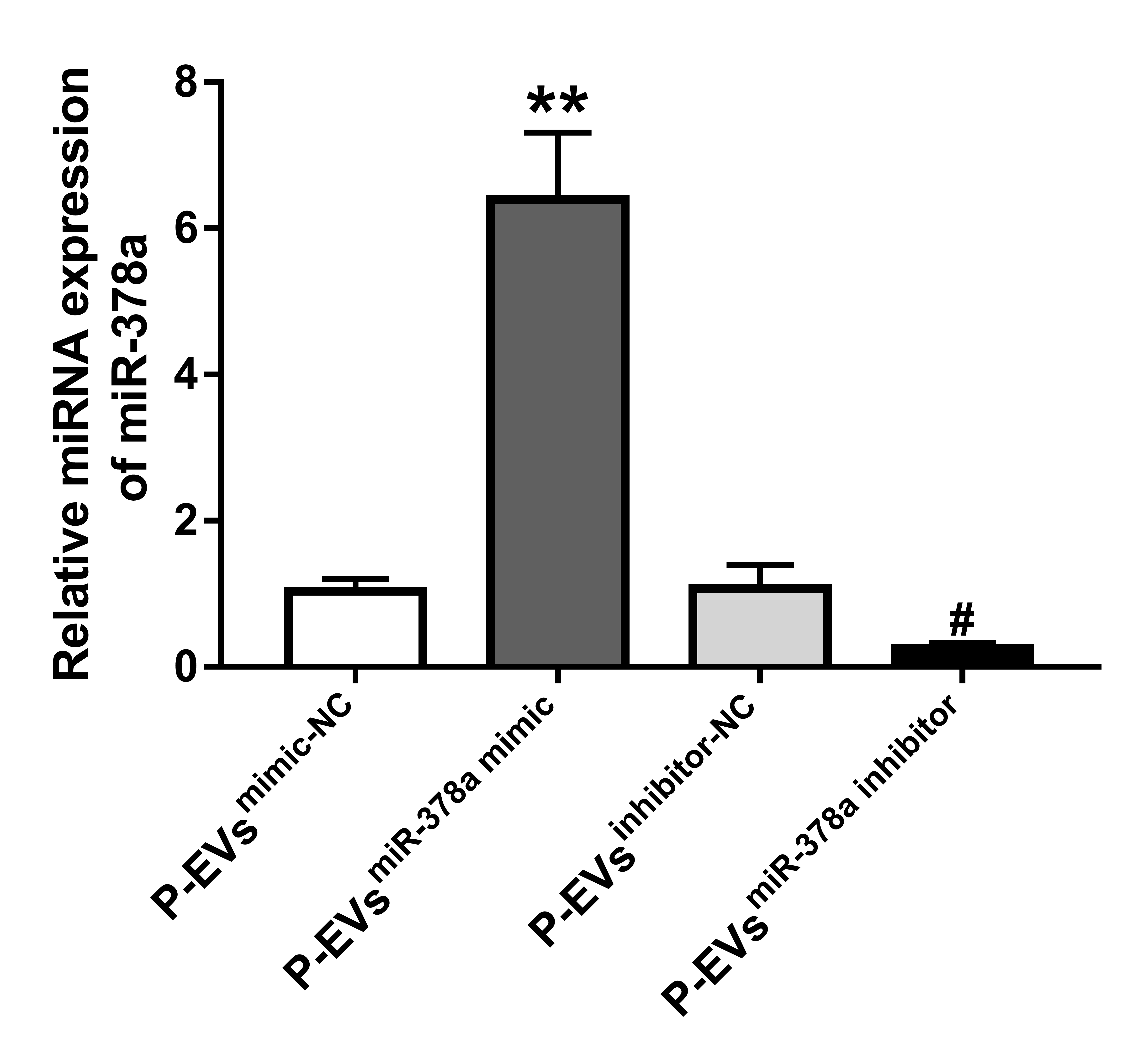


**Fig. S3.** RT-PCR analysis of miR-378a expression in ECs incubated with P-EVs^mimic-NC^, P-EVs^miR-378a mimic^, P-EVs^inhibitor-NC^, P-EVs^miR-378a inhibitor^ for 48 h (*n* = 3). ***P* < 0.01 vs. the P-EVs^mimic-NC^ group; **^#^***P* < 0.05 vs. the P-EVs^inhibitor-NC^ group.
