# Supplementary material for: Periodontitis‐compromised dental pulp stem cells secrete extracellular vesicles carrying miRNA‐378a promote local angiogenesis by targeting Sufu to activate the Hedgehog/Gli1 signalling
Source: Cell Prolif. 2021 Mar 23;54(5):e13026. doi: 10.1111/cpr.13026 (PMC8088471; doi:10.1111/cpr.13026)
Supplement: Supplementary file 4 — Supplementary Material [file CPR-54-e13026-s002.docx]

**Periodontitis-compromised dental pulp stem cells secrete extracellular vesicles carrying miRNA-378a promote local angiogenesis by targeting Sufu to activate the Hedgehog/Gli1 signaling**

**Supplemental experimental procedures**

1. **Isolation and identification of P-DPSCs**
   1. ***Cell isolation and culture***

Patients who had (i) at least one tooth with full or partial pulp vitality that was to be extracted due to irreversible periodontitis and (ii) at least one other periodontally healthy tooth that was to be extracted due to nonfunctional or impacted reasons (often the third molars) were asked to donate their teeth for cell isolation. If cell isolation failed for one tooth, the participant was excluded. The present research protocol was approved by the Ethics Committee of the Stomatological Hospital of Fourth Military Medical University (FMMU, 201203), and informed consent was signed by all the subjects. Finally, 5 pairs of teeth were obtained from 5 systemically healthy donors (male. 2; female. 3; age. 24~41 years); H-DPSCs and P-DPSCs were successfully isolated from pulp tissues of 11 teeth (periodontally healthy teeth, 5; periodontitis teeth, 6) in parallel. 5 teeth belonging to 2 donors were excluded due to culture failure.

The isolation of DPSCs were performed according to our previously reported methods.^1^ In brief, the collected teeth were rinsed with sterile phosphate-buffered saline (PBS; Corning, NY, USA), then the teeth were sawed to expose the pulp cavity, and the pulp tissues were collected and cut into small pieces. Afterwards, the tissue tips were incubated with type I collagenase (3 mg/mL; DIYIBio; Shanghai, China) for 1 h at 37°C. After that, the tissue tips were centrifuged and resuspended in α-minimum essential medium (α-MEM; Gibco BRL, Grand Island, NY, USA) containing 10% fetal bovine serum (FBS; Sijiqing, Hangzhou, China) and 1% penicillin-G/streptomycin (Invitrogen, Carlsbad, CA, USA), then seeded into 12-well plates (Corning, Lowell, MA, USA) at an atmosphere of 5% CO_2_ at 37 °C. The limiting dilution technique was used for DPSC purification. DPSCs at passages 3-5 (P3–P5) were used for the following investigation.

***1.2. Flow cytometry***

Flow cytometry assay was utilized to identify the immunophenotypes of P-DPSCs as the previously reported methods.^2^ In brief, DPSCs (P3) were collected and washed twice with adequate PBS containing 3% FBS. After that, the cell suspension was divided into sterile Eppendorf (EP) tubes (Axygen, Tewksbury, MA, USA) and then incubated in dark with monoclonal antibodies against human CD90, CD105, CD146, CD34, CD45, and CD31 (all from eBioscience, San Diego, CA, USA) for 1 h at 4°C. Subsequently, DPSCs were washed twice with PBS, then resuspended in 400 μL PBS and detected with the flow cytometry (Beckman Coulter, Fullerton, CA, USA).

***1.3. Cell differentiation assay***

To identify their multiple differentiation potential, P-DPSCs (P3) were collected and cultured in complete α-MEM, then were seeded into 6-well plates (2 × 10^5^ cell/per well). After reaching 80% confluence, the P-DPSC culture medium was replaced with osteogenic differentiation medium, adipogenic differentiation medium, and chondrogenic differentiation medium (all from Cyagen, Guangzhou, China). After induction for 21 d (for osteogenic differentiation and adipogenic differentiation) or 28 d (for chondrogenic differentiation), the cells were fixed with paraformaldehyde (4%) for 30 min, and then stained with Alizarin Red S (for osteogenic differentiation), Oil Red O (for adipogenic differentiation), and Alcian blue staining (for chondrogenic differentiation), respectively.

**Appendix S1 References**

1. Sun HH, Chen B, Zhu QL, Kong H, Li QH, Gao LN, et al. Investigation of dental pulp stem cells isolated from discarded human teeth extracted due to aggressive periodontitis. *Biomaterials*. 2014;35:9459–72.

2. Li X, He XT, Kong DQ, Xu XY, Wu RX, Sun LJ, Tian BM, Chen FM. M2 Macrophages Enhance the Cementoblastic Differentiation of Periodontal Ligament Stem Cells via the Akt and JNK Pathways. *Stem Cells*. 2019;37(12):1567-1580.

**Supplemental Tables**

**Table S1.** Primer sequences of miRNAs used in this study.

| Gene name | Primer sequence (5’-3’) |
| --- | --- |
| Sufu | F: CTGCACTGAAGAGCTACACTC  R: CTCAAATATGGTCTCTCCCCTC |
| hsa-miR-378a | F: ACTGGACTTGGAGTCAGAAGGC |
| has-miR-595 | F: GAAGTGTGCCGTGGTGTGTCT |
| has-miR-7844-5p | F: GCAAAACTAGGACTGTGTGGTGTA |
| has-miR-3692-5p | F: CCTGCTGGTCAGGAGTGGATA |
| has-miR-6833-5p | F: GTGTGGAAGATGGGAGGAGAAA |
| U6 | F: AACGAGACGACGACAGAC  R: GCAAATTCGTGAAGCGTTCCATA |

**Table S2.** Oligonucleotide Sequences for miR-378a and si-RNA(Sufu)

| Gene name | Primer sequence (5’-3’) |
| --- | --- |
| si-Sufu-homo-683 (si-Sufu #1) | F: GCCCUUUGGAUAACAGUGATT  R: UCACUGUUAUCCAAAGGGCTT |
| si-Sufu-homo-1363 (si-Sufu #2) | F: GGACGGCACUUUACAUAUATT  R: UAUAUGUAAAGUGCCGUCCTT |
| si-Sufu-homo-1518 (si-Sufu #3) | F: GUUGGAGGAUUUAGAAGAUTT  R: AUCUUCUAAAUCCUCCAACTT |
| has-miR-378a mimic | F: ACUGGACUUGGAGUCAGAAGGC  R: CUUCUGACUCCAAGUCCAGUUU |
| mimic NC | F: UUCUCCGAACGUGUCACGUTT  R: ACGUGACACGUUCGGAGAATT |
| has-miR-378a inhibitor | R: GCCUUCUGACUCCAAGUCCAGU |
| Inhibitor NC | R: CAGUACUUUUGUGUAGUACAA |
